# First-Time Submassive Pulmonary Embolism Likely Caused by Testosterone-Enhancing Supplement

**DOI:** 10.7759/cureus.25103

**Published:** 2022-05-18

**Authors:** Hazem Alakhras, Brent R Yelton, Hamza Beano

**Affiliations:** 1 Internal Medicine, Oakland University William Beaumont School of Medicine, Rochester, USA; 2 Urology, Beaumont Hospital, Royal Oak, USA

**Keywords:** pulmonary thrombectomy, unprovoked pulmonary embolism, prophylactic and therapeutic anticoagulation, herbal supplement adverse event, polycythemia, deep vein thrombosis (dvt), erectile dysfunction, fenugreek, testosterone-induced thrombosis

## Abstract

Pulmonary embolism (PE) is a potentially fatal occurrence with a broad spectrum of risk factors. A 75-year-old male presented to the emergency room with five days of shortness of breath, back pain, and hemoptysis. A CT angiogram demonstrated bilateral pulmonary emboli with a larger thrombus on the right, as well as signs of right heart strain. The patient was started on IV heparin and ultimately underwent a successful embolectomy. Evaluation to determine the underlying etiology of this patient's first-time PE was performed to further stratify his risk of recurrence and the length of anticoagulation required. The provoking factor for his PE was initially unclear as he lacked any risk factors such as recent surgeries, periods of immobility, or previous diagnosis of malignancy. The patient was noted to be on an erectile dysfunction supplement called “Eroxin,” and he had been taking it for the past six months. Eroxin contains an ingredient called fenugreek, which is believed to enhance testosterone levels by inhibiting aromatase and 5-alpha-reductase activity. Fenugreek has previously been associated with the formation of PEs, and likely contributed to the PE in this patient. This is likely due to testosterone-induced polycythemia and increased platelet aggregation. This case highlights the concern around supplements as their ingredients are poorly regulated and occasionally found to be tainted with unlisted ingredients. This also highlights the importance of gathering a complete supplement history from patients as their use can lead to serious illness. Lastly, it encourages considering testosterone use as a potential thrombogenic risk factor.

## Introduction

Pulmonary embolism (PE) is a serious and potentially fatal condition accounting for 100,000 deaths annually. Overall, the incidence in the United States is around 100 cases per 100,000 individuals, with a slightly higher incidence in males compared to females, and in the elderly over the age of 75 years [1]. A high index of suspicion is needed as the presentation of PE can vary from no or insidious signs to sudden death. Common presenting symptoms include tachycardia, dyspnea, signs of deep vein thrombosis (DVT), pleuritic chest pain, and cough with hemoptysis [2].

Due to the variability in the severity of presentation of PE, there are different imaging modalities and serum tests that can be utilized. Initial evaluation to determine the pretest probability of PE can be done using scoring systems such as the Wells criteria and the pulmonary embolism rule-out criteria (PERC). These criteria can be used to determine the utility and necessity of further testing such as CT pulmonary angiography (CTPA) and serum D-dimer. A filling defect in the pulmonary arteries on CTPA is diagnostic [2]. Further workup involves stratification based on the presence or absence of right ventricular (RV) dysfunction, myocardial necrosis, and hypotension. This is then further categorized into non-massive PE, submassive PE, and massive PE from lowest to highest risk as a way to guide treatment [3].

In those with high suspicion of PE on initial assessment, treatment involves oxygenating and stabilizing the patient. Once a diagnosis has been made, treatment depends on severity. In patients with a non-life-threatening presentation, treatment involves anticoagulation with subcutaneous heparin or fondaparinux, or direct oral anticoagulants (DOACs) like rivaroxaban or apixaban. In patients with a life-threatening PE, reperfusion treatments such as thrombolysis or embolectomy may be performed depending on a patient's bleeding risk [2]. Following stabilization in the hospital, patients are started on three months of DOACs in the outpatient setting. Patients with an unprovoked PE or increased risk factors should be considered for indefinite anticoagulation [2].

There are some patient factors and medications that increase the risk of DVT and thus PE in patients. These include increased age, malignancy, pregnancy, obesity, venous stasis, and heritable hypercoagulable disorders. Certain medications such as estrogen-containing contraceptives have been shown to increase the risk of thrombosis [2]. Recent literature has been equivocal on the thrombogenicity of testosterone-replacement therapy (TRT). However, numerous case reports document the relationship between the use of TRT and hypercoagulable states that can lead to PE [4,5].

There has been an increase in interest in aging men using herbal supplements as a way to boost their sexual drive and help alleviate erectile dysfunction symptoms. Fenugreek (*Trigonella foenum-graecum*) is a plant extract that is a common ingredient found in these testosterone-enhancing supplements. Its mechanism of action is theorized to inhibit 5-alpha-reductase and aromatase, thus increasing the amount of testosterone and dihydrotestosterone [6]. It is proposed that testosterone can lead to hypercoagulable states by two major mechanisms. First, by increasing erythropoietin levels, polycythemia can increase the risk of PE due to increased viscosity and stagnant blood flow [7]. Second, testosterone has been linked with an increased expression of thromboxane A2 receptors on platelets, thus potentially playing a role in thrombosis [8].

## Case presentation

Signs and symptoms

A 75-year-old Caucasian male with a medical history significant for chronic lower-extremity edema and self-reported erectile dysfunction presented to the emergency department with the chief complaints of shortness of breath and back pain. He reported that the shortness of breath started five days prior and his right upper back pain is worse when he leans backward or takes deep breaths. He described the back pain as excruciating and reported only mild relief with ibuprofen. On the day of admission, he experienced a subjective fever and hemoptysis. The patient had chronic lower-extremity edema and discoloration due to stasis dermatitis. He reported that his left calf had been intermittently tender over the last five days and that it seemed more swollen than baseline on the day of admission. He had been prescribed furosemide for the edema but it did not provide relief, and he had a pending lower-extremity ultrasound. The patient denied any recent travel, surgery, immobility, or history of personal or familial thrombophilias or DVTs. Upon further questioning, he did note that he started taking a supplement called Eroxin daily for the previous six months to help with his sexual dysfunction. He was a previous five-pack-year smoker, but quit 33 years ago, had a negative colonoscopy screening five years ago, and is non-obese. He denied any chest pain, orthopnea, paroxysmal nocturnal dyspnea, weight loss, and night sweats.

On physical exam, the patient had a 1/6 systolic murmur present with tachycardia, but regular rhythm. He also had normal breath sounds, but increased respiratory rate and effort. His lower extremities were significant for 1+ pitting edema and erythema. In the emergency department, the patient was hypertensive at 184/90 mmHg, tachycardic at 104 beats per minute, tachypneic at 32 breaths per minute, afebrile, and saturating at 93% on room air.

Labs and imaging

**Figure 1:**
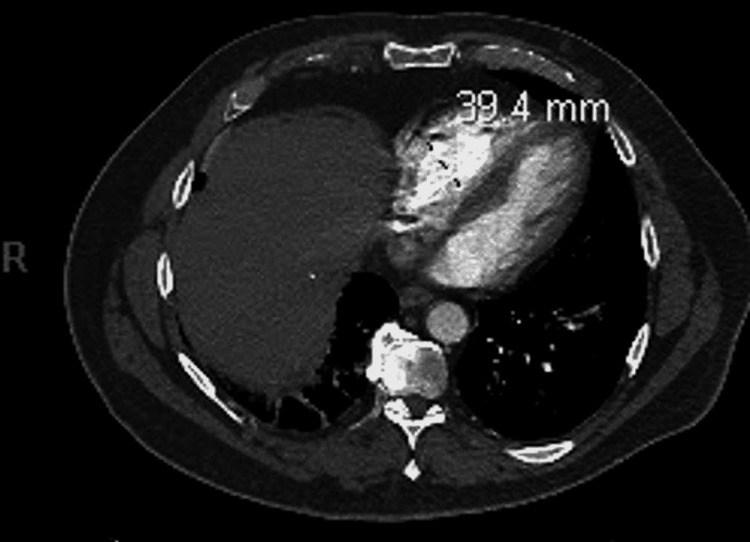
CT pulmonary angiography showing bilateral pulmonary emboli, with a larger thrombus on the right.

His initial complete blood count (CBC) was remarkable for mild leukocytosis of 11.3 bil/L and polycythemia of 17.4 g/dL with a hematocrit of 54%. Labs six months prior showed hemoglobin of 15 g/dL and hematocrit of 46%. Other labs showed an international normalized ratio (INR) of 1.1 and partial thromboplastin time (PTT) at 27 seconds. His basic metabolic panel (BMP) was within normal limits. The patient had a normal brain natriuretic peptide (BNP) of 20 pg/mL, troponin of 0.02 ng/mL, and he was negative for coronavirus disease 2019 (COVID-19). An ECG demonstrated sinus tachycardia with normal axis and rhythm, and no ST elevation or depression. A chest X-ray showed cardiomegaly, minimal basilar lung atelectasis, normal pulmonary vasculature, and no consolidations. Based on the patient's initial presentation, there was a high suspicion of PE, so a CTPA was ordered. This showed bilateral pulmonary emboli with a larger thrombus on the right (Figure 1). There was also evidence of RV strain with an RV to left ventricle (LV) ratio greater than one. After the thrombectomy was performed, a transthoracic echocardiogram showed a left ventricular ejection fraction at 60%, mild aortic stenosis, and mild aortic regurgitation. Lower extremity duplex ultrasound was also done and was negative for DVT bilaterally.

Management

Based on the signs of right heart strain, and normal troponin/BNP levels, the patient was stratified as a submassive PE with intermediate risk. A multidisciplinary decision was made to escalate care. The therapy consisted of catheter-directed thrombectomy via FlowTriever (Inari Medical, Irvine, California) and immediate anticoagulation. The patient was started on an IV heparin drip, placed on 4 L of oxygen, and the thrombectomy was completed the following morning without any complications. Following the procedure, the patient improved symptomatically and was eventually transitioned from IV heparin drip to rivaroxaban to continue at home. The patient was discharged with plans for outpatient cardiology follow-up for management of his DOAC. He was also advised to discontinue taking this or other supplements, as it was considered the most likely etiology of this PE.

## Discussion

There are multiple causes of PE or DVT. After a patient is diagnosed and stabilized, it is important to consider the underlying etiology to determine the risk of recurrence and length of anticoagulation. Our patient lacked strong risk factors, as he had no recent surgeries or significant traumas [9]. He also did not have any moderate risk factors such as a history of congestive heart failure or a history of taking estrogen-containing medications [9]. The patient had no history of malignancies, was up to date on his colonoscopy screening, and did not meet guidelines for lung cancer screening [10]. Although an underlying malignancy cannot be ruled out as a cause, there were no clinical signs pointing towards an undiagnosed malignancy in our patient. Thrombophilia, such as factor V Leiden, is also unlikely as he had no family history of venous thromboembolism, and those with hereditary thrombophilias generally present at a younger age. While he is considered of older age, he does not fall under any other weak risk factors such as obesity or limited mobility [9].

Without an obvious source for this patient's PE, other risk factors were carefully considered. One remaining area of interest was to investigate the sexual-enhancement supplement that he started six months ago. After extensive research, a website was found that listed the active ingredients like fenugreek, *Mucuna pruriens*, and *Malus pumila* [11]. Fenugreek, which is theorized to increase testosterone levels and can explain our patient’s polycythemia, was regarded as high on the differential for causing our patient’s PE. While testosterone levels were not measured for our patient, there are case reports linking this testosterone-enhancing supplement with thromboembolism. One case report had a similar patient without risk factors who also used a form of TRT that contained fenugreek as its active ingredient; like our patient, he too had a first-time bilateral PE [4]. An additional case report describes another patient without risk factors and negative thrombophilia screenings who directly used exogenous testosterone that ultimately led to a first-time bilateral PE [5]. Based on this evidence, the lack of other risk factors in our patient, and the timing of our patient’s supplement use, we have a high suspicion of Eroxin provoking PE.

Supplements are often unregulated and can predispose to disease. There is uncertainty about their safety profiles and effectiveness [12]. Contamination is a major issue, as 83 different sexual-enhancement supplements were found to be tainted with hidden ingredients since 2020 [13]. In addition, manufacturing can be inconsistent, since a recent study found many testosterone-enhancing supplements had supratherapeutic doses of vitamins and other minerals [14]. With that said, supplements should be cautiously considered by providers as ingredients, such as fenugreek, in this case, may play a role in the disease process of the patient.

Given the inconsistencies and uncertainties with supplements, supplements should be carefully assessed during patient evaluation. A 2015 study of previously hospitalized patients found that 60% of patients reported daily supplement use. This study showed that only 20% of patients were asked about supplement use at the time of admission and only 6% of those had it correctly documented [15]. These findings were supported by another study that found only 33% of patients disclosed their supplement use to their provider [16]. This points toward hesitancy in patients reporting their supplement use as well as some inconsistent interviewing by physicians.

## Conclusions

Based on our patient’s absence of risk factors and the potential hypercoagulability associated with testosterone use, our patient’s PE was likely caused by his fenugreek-containing supplement. This emphasizes the value of keeping a wide differential and warrants further research that studies testosterone's potential thrombogenicity. Nevertheless, there is a real concern with using this and other similar supplements that are poorly regulated, as they can be misleading and potentially dangerous. This case exemplifies the significance of taking a thorough history of supplement use in patients and the importance of cautioning patients on their potential risks.
